# Short-term comparison of switching to faricimab from other anti-VEGF agents in neovascular age-related macular degeneration patients: A retrospective study

**DOI:** 10.1097/MD.0000000000042002

**Published:** 2025-04-25

**Authors:** Dingxi Liu, Chang Li, Lin Cui, Sheng Li

**Affiliations:** a Department of Ophthalmology, The Third People’s Hospital of Dalian, Dalian Medical University, Dalian, China.

**Keywords:** epithelium, macular degeneration, visual acuity

## Abstract

In order to evaluate the short-term outcomes of switching to faricimab from other anti-vascular endothelial growth factor (VEGF) agents in Chinese patients with neovascular age-related macular degeneration (nAMD). This was a retrospective, observational study involving patients with nAMD who had insufficient response to previous anti-VEGF therapy and were switched to Faricimab. Best-corrected visual acuity, central macular thickness, and pigment epithelium detachment (PED) changes were recorded at baseline and after one month of treatment. Data were analyzed using paired t-tests to compare outcomes before and after the switch. This study included 35 eyes from 35 patients (mean age 69.74 ± 11.22 years) who were switched to Faricimab after an average of 6.27 ± 3.41 prior anti-VEGF injections for nAMD. While best-corrected visual acuity showed no significant improvement after one month (*P* = .06), significant reductions were observed in mean central macular thickness (*P* < .001), PED height (*P* < .001), PED volume (*P* < .001), presence of subretinal fluid (*P* = .03), and intraretinal fluid (*P* = .04). Additionally, the presence of PED decreased from 60% at baseline to 45.71% after one month (*P* = .02). No new safety concerns were identified during the study period. Switching to faricimab from other anti-VEGF agents resulted in significant short-term improvements in both visual and anatomical outcomes, including reduced central macular thickness, pigment epithelium detachment, and subretinal and intraretinal fluid. These findings suggest that Faricimab may offer a beneficial alternative for patients with an insufficient response to prior anti-VEGF therapies. Long-term follow-up studies are necessary to confirm the durability and long-term benefits of this treatment.

## 1. Introduction

Neovascular age-related macular degeneration (nAMD) is a leading cause of visual impairment among the elderly worldwide.^[1,2]^ A defining characteristic of nAMD is the formation of pigment epithelial detachment (PED), which occurs in about 30% to 80% of nAMD cases.^[3]^ This detachment of the retinal pigment epithelium (RPE) from Bruch’s membrane is caused by fluid buildup (fluid in the subretinal [SRF] and/or intra-retinal space [IRF]) and abnormal vascular growth, resulting in various clinical presentations. Vascular endothelial growth factor (VEGF) is a key regulator in the process of macular neovascularization (MNV) and the modulation of vascular permeability.^[4]^ The introduction of anti-VEGF agents has revolutionized the management of nAMD, offering significant improvements in visual outcomes,^[5]^ including Ranibizumab, Aflibercept, and Conbercept. However, a subset of patients exhibits a suboptimal response to conventional anti-VEGF therapies even after receiving consecutive monthly injections, necessitating alternative treatment strategies.^[6,7]^ Consequently, continuous efforts have been directed toward enhancing therapeutic efficacy, including the development of new drugs.

Faricimab (Vabysmo, Roche, Basel, Switzerland), a bispecific antibody developed for the treatment of nAMD, has been shown to be effective in controlled clinical studies. The regulatory approval of Faricimab in China was granted following evidence from pivotal trials such as the TENAYA and LUCERNE studies. These trials demonstrated that Faricimab, when administered every 8 weeks, was non-inferior to aflibercept in terms of visual acuity outcomes and offered a longer dosing interval, which may improve patient compliance and quality of life. The TENAYA and LUCERNE studies involved a diverse range of patients, providing strong support for the use of Faricimab in nAMD, and these results were integral to its approval by regulatory agencies, including in China.^[8,9]^ Preliminary studies have suggested that Faricimab may provide superior outcomes in certain patients, particularly those with an inadequate response to other anti-VEGF agents.^[10]^ Despite the growing body of literature on faricimab switching, no published studies to date have specifically evaluated this treatment strategy in Chinese patients with nAMD.

This study aims to evaluate the short-term visual outcomes, such as best-corrected visual acuity (BCVA), and anatomical outcomes, including central macular thickness (CMT) and pigment PED changes, in Chinese patients with nAMD who were switched to faricimab from other anti-VEGF agents due to inadequate response. Understanding the potential benefits and safety of Faricimab in this context is crucial for optimizing treatment strategies for nAMD.

## 2. Methods

### 2.1. Study design and participants

This retrospective, observational study included Chinese patients with nAMD who were switched to faricimab after demonstrating an inadequate response to other anti-VEGF therapies, prior treatment with at least three injections of anti-VEGF agents, and documented evidence of persistent fluid or stable/increasing central retinal thickness. This study conducted between March 2024 and July 2024 at the Department of Ophthalmology, The Third People’s Hospital of Dalian, China. This study was approved by the Ethics Committee of The Third People’s Hospital of Dalian (ID number: 2024-106-001) and adhered to the tenets of the Declaration of Helsinki. Written informed consent was not obtained because the study was retrospective. The need for informed consent was waived by the Ethics Committee of The Third People’s Hospital of Dalian.

The inclusion criteria for the study were: (1) a confirmed diagnosis of MNV secondary to nAMD; (2) evidence of recurrent fluid following treatment with other anti-VEGF; (3) a decision to switch to Faricimab based on one or more of the following: inadequate visual acuity improvement or stability, persistent fluid presence, or challenges in extending treatment intervals; and (4) completion of a one-month observation period after the switch to faricimab.

The exclusion criteria for the study were: (1) history of treatments with photodynamic therapy, (2) any other retinal or optic nerve disease, (3) polypoidal choroidal vasculopathy, given its distinct features compared to nAMD; and (4) presence of inflammatory or hereditary diseases that may contribute to MNV.

Patients received intravitreal injections of faricimab (6 mg) at baseline (day 0), and subsequently follow-up at days 30.

BCVA was tested at 5 m using Standard Logarithm Visual Acuity chart in decimal notations. Other ocular examinations including fundus examination, and Spectral-Domain Optical Coherence Tomography imaging (Spectralis, Heidelberg Engineering, Heidelberg, Germany) utilizing high-density scans. SRF was identified as areas of round or oval hypo-reflective regions with well-defined margins, located between the RPE and the neuroretina. IRF was characterized by the expansion of retinal bands relative to those in a healthy retina. PED were defined as localized protrusions of the reflective RPE band, with an underlying space that appears optically clear or moderately reflective. nAMD is subcategorized into type 1, type 2, and type 3 MNV as determined by fluorescein angiography (FA), indocyanine green angiography (ICGA), and optical coherence tomography (OCT).

The spectral-domain optical coherence tomography measurements, as assessed by the same trained grader, encompassed the following: (a) PED metrics, including maximum height, and total volume; (b) retinal metrics, such as mean retinal thickness; (c) presence of SRF, IRF, or PED. Patients eligible for the study were diagnosed with nAMD based on a comprehensive evaluation, including clinical examination, OCT, and fundus photography. The diagnosis was confirmed by retinal specialists in accordance with the current guidelines for nAMD. A Cirrus HD-OCT 5000 imager (Carl Zeiss, Germany) was utilized to assess retinal morphological changes in the macular region, quantify PED volume. The RPE analysis protocol was applied with 6-mm horizontal and vertical line scans, with a scanning depth of 2 mm and an adjustable scanning length of 6 to 9 mm, depending on the lesion size. Images were acquired at a resolution of 512 × 128 pixels. A 5-line raster scan (0.25 × 6 mm) was performed at the highest point of the PED, where its vertical height was manually measured using the built-in caliper tool on the OCT monitor. An advanced RPE analysis system was employed to manually select pre- and post-treatment PED images, with a 5-mm circular volume chosen for comparative analysis.

Ocular complications (i.e., RPE tears, uveitis, endophthalmitis, and geographic atrophy) and systemic adverse events were tracked in this study.

### 2.2. Statistical analysis

Continuous data were summarized using means and standard deviations (SD), whereas categorical variables were described with counts and percentages. Paired *t* tests were conducted to compare baseline and follow-up outcomes, including changes in BCVA, central retinal thickness, PED height, and PED volume. Chi-square tests were employed to evaluate the associations between categorical variables. Statistical analyses were performed with Statistical Product and Service Solutions version 22 (IBM SPSS, Armonk, New York). A *P*-value < .05 was considered statistically significant.

## 3. Results

The study included 35 eyes from 35 patients with nAMD (mean age 69.74 ± 11.22 years), including 12 females, all of whom were switched to faricimab due to an inadequate response to other anti-VEGF agents (Table 1). In terms of MNV classification, 16 patients had type I, 12 had type II, and 7 had type III macular neovascularization.

**Table 1:** Characteristics of patients or eyes before and after switching (n = 35).

Variables	Baseline	1 month	*P*
Age (years), mean (SD)	69.74 ± 11.22		
Gender, female, n (%)	12 (34.3)		
Eyes, right, n (%)	13 (37.1)		
Pre injection	6.27 ± 3.41		
Best corrected visual acuity, LogMAR, mean (SD)	0.38 ± 0.12	0.29 ± 0.25	.06
Central macular thickness, µm, mean (SD)	511.78 ± 110.29	351.48 ± 101.23	<.001
Presence of SRF, n (%)	20 (57.14)	11 (31.42)	.03
Presence of IRF, n (%)	16 (45.71)	8 (22.85)	.04
Presence of PED, n (%)	25 (60)	16 (45.71)	.02
PED height, µm, mean (SD)	331.56 ± 112.23	209.65 ± 89.45	<.001
PED volume, nl, mean (SD)	2137.11 ± 966.23	1473.23 ± 754.12	.002

IRF = intraretinal fluid, logMAR = logarithm of the minimum angle of resolution, PED = pigment epithelial detachments, SD = standard deviation, SRF = subretinal fluid.

All patients had previously received 6.27 ± 3.41 injections of other anti-VEGF agents before switching to faricimab (Table 1). Among the enrolled patients, 12 were previously treated with aflibercept, 14 with ranibizumab, and 9 with conbercept before switching to FARICIMAB. The BCVA results were converted to the logarithm of the minimum angle of resolution. At the one-month follow-up, mean BCVA improved from 0.38 ± 0.12 at baseline to 0.29 ± 0.25, though this change was not statistically significant (*P* = .06). However, the mean CMT decreased from 511.78 ± 110.29 to 351.48 ± 101.23 (*P* < .001) after injection. Additionally, PED height was changed from 331.56 ± 112.23 to 209.65 ± 89.45 (*P* < .001), and PED volume decreased from 2137.11 ± 966.23 to 1473.23 ± 754.12 (*P* < .001). Furthermore, SRF on OCT at baseline was present in 20 eyes (57.14%) and in 11 eyes (31.42%) after 1 month injection (*P* = .03). IRF on OCT at baseline was observed in 16 eyes (45.71%) and in 8 eyes (22.85%) after injection (*P* = .04). Additionally, the presence of PED was seen in 25 eyes (60%) at baseline and there were 16 eyes (45.71%) of patients exhibited PED (*P* = .02) (Table 1).

No significant adverse events were reported after one-month Faricimab injection among all subjects with nAMD.

### 3.1. Case presentation

**Figure 1.**
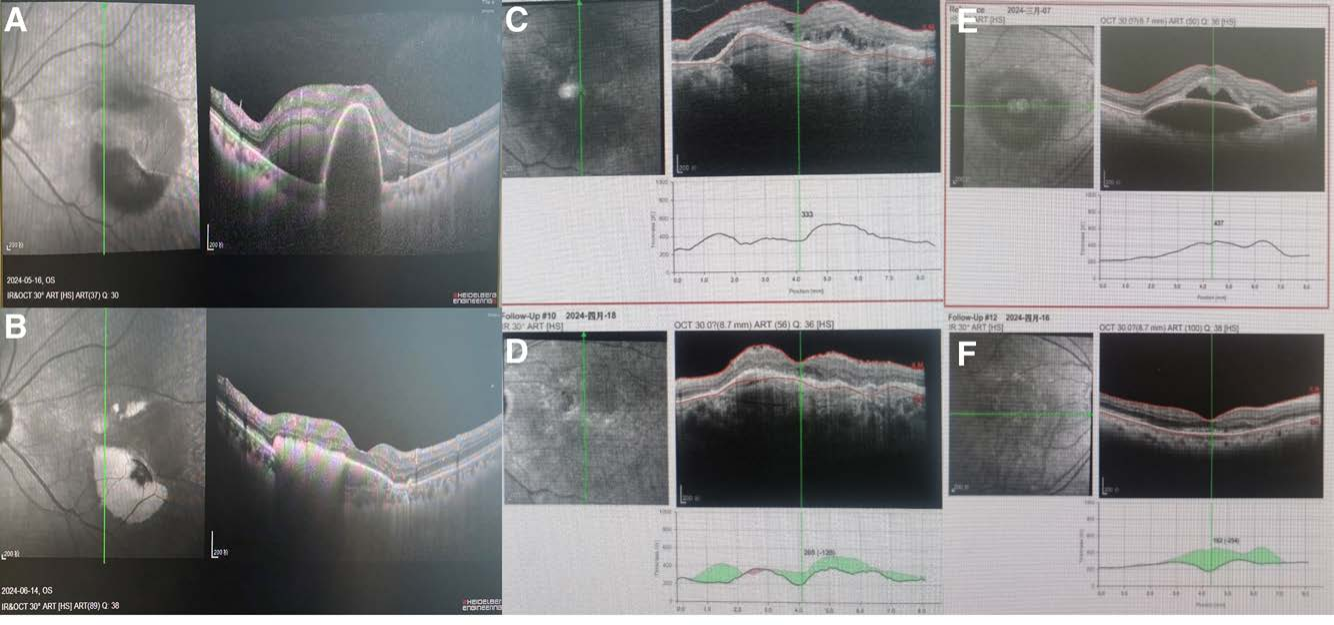
Three representative cases of switching from other anti-VEGF agents to faricimab in a Chinese patient with nAMD. Optical coherence tomographic scans going through the fovea at baseline and one-month after injection. After switching to faricimab, the patient (A–F) decreases in CMT and the height of the pigment epithelial detachment were observed. CMT = central macular thickness, nAMD = neovascular age-related macular degeneration, VEGF = vascular endothelial growth factor.

The following case study provides an example of the anatomical outcomes after switching to faricimab from other anti-VEGF agents (Fig. 1). A 70-year-old man diagnosed with nAMD in 2024 required frequent intravitreal aflibercept injections, receiving a total of four injections. After the latest intravitreal aflibercept injection on April 18, 2024, she was switched to faricimab on May 16, 2024 due to insufficient resolution of pigment epithelial detachment (Fig. 1A and B). Furthermore, a 78-year-old woman diagnosed with nAMD in 2024 switching to Faricimab from other anti-VEGF agents also received PED reduction (Fig. 1C and D). Another 74-year-old man diagnosed with nAMD in 2022, who transitioned to faricimab from other anti-VEGF agents, also demonstrated a significant reduction in PED (Fig. 1E and F).

## 4. Discussion

Our findings suggest that faricimab is a promising alternative for Chinese patients with nAMD who show inadequate response to other anti-VEGF therapies. The significant improvements in anatomical outcomes, as seen in BCVA and PED reduction, support Faricimab’s dual-targeting mechanism and align with emerging evidence for its efficacy. To our knowledge, this is among the first studies to directly compare outcomes following the switch to faricimab from other anti-VEGF agents in a Chinese patients within a routine clinical practice setting.

Faricimab, a recently approved bispecific antibody in China, targets both VEGF-A and angiopoietin-2 (Ang-2), offering a dual mechanism of action in the treatment of nAMD and diabetic macular edema. By simultaneously inhibiting these critical pathways in the pathogenesis of MNV, faricimab not only addresses the angiogenic drivers but also promotes vascular stability, thereby offering a potentially enhanced therapeutic approach for managing PED.^[11,12]^ The lack of substantial visual improvement following the transition to faricimab during short-term follow-up, as noted in our study, is consistent with previous findings.^[13–16]^ These studies have postulated that the suboptimal gain in BCVA may be attributed to irreversible photoreceptor damage present at the time of the switch.

These results underscore the potential of faricimab to address the unmet needs of patients with refractory nAMD, particularly in the short-term. Faricimab helps restore vascular stability and promote vessel maturation by inhibiting Ang-2,^[11]^ and may more effectively restrict inflammatory mechanisms linked to vascular permeability and fluid accumulation in the sub-RPE space.^[17]^ Although a three-month period may have been insufficient for faricimab to fully demonstrate its potential, both treatments achieved significant reduction in CMT, PED height, PED volume as well as presence of IRF, SRF, and PED compared to baseline at 1 month. The pronounced and swift reduction in PED volume observed in our study could potentially contribute to favorable long-term functional outcomes. Prior study has demonstrated a significant association between early resolution of PED and improved long-term visual acuity, underscoring the importance of prompt PED management.^[18]^ Faricimab’s dual inhibition of VEGF-A and Ang-2 represents a promising advancement for treating nAMD. Unlike traditional therapies that only target VEGF-A, faricimab’s additional Ang-2 inhibition may offer enhanced benefits by reducing vascular instability and inflammation, which are implicated in persistent fluid and inflammation in nAMD. Studies, including TENAYA and LUCERNE,^[19]^ suggest that this dual-action approach may lead to better outcomes in patients who are refractory to VEGF-A monotherapy, with observed reductions in CMT and PED, which was consistent with our findings. Generally, these outcomes encourage further investigation into Ang-2’s role, potentially shaping future treatment options for more durable control in nAMD.

However, there were some limitations needing cautious. First, this is a non-randomized, uncontrolled observational design inherently restricts our capacity to rigorously compare the efficacy of Faricimab against other anti-VEGF therapies. Second, the single-center design and relatively small sample size introduce potential selection and investigator bias, thereby limiting the broader applicability of our findings. Additionally, the short duration of follow-up constrains the assessment of Faricimab’s long-term efficacy. Extended follow-up data would be essential to further substantiate the durability and effectiveness of faricimab in this patient population. These preliminary results provide a promising outlook on faricimab as an alternative in cases of refractory nAMD. While one-year prospective studies on Faricimab exist, further controlled studies in Chinese populations with extended follow-up are warranted to validate these findings. Another limitation is the reliance on short-term observational data; while OCT parameters provide objective anatomical assessments, additional functional measures and longer follow-up are needed for a more comprehensive evaluation. As a descriptive and observational study, we did not measure clinical outcomes or use objective data such as laboratory tests, imaging, or physiological measurements. Additionally, a major limitation of our study is the lack of a control group, which restricts our ability to draw definitive conclusions regarding causality. As an observational study, our design allows us to identify associations, but without a control group, we cannot exclude the possibility of confounding factors influencing the results. This limits the generalizability of our findings and the strength of the causal inferences that can be made. Future studies that include a control group would be important to confirm these associations and to establish stronger evidence of causal relationships.

## 5. Conclusions

Switching from pre anti-VEGF injection to Faricimab yields significant anatomical benefits in eyes with nAMD, with both treatments proving to be effective short-term options in China. Further research with a larger sample size, extended observation periods, and standardized injection regimens is necessary to identify the optimal switching strategy from anti-VEGF in routine clinical practice.

## Acknowledgments

The authors thank all the people who had participated in this study.

## Author contributions

**Conceptualization:** Dingxi Liu, Sheng Li.

**Formal analysis:** Dingxi Liu, Sheng Li.

**Investigation:** Sheng Li.

**Supervision:** Sheng Li.

**Writing – original draft:** Dingxi Liu, Chang Li, Lin Cui, Sheng Li.

**Writing – review & editing:** Dingxi Liu, Chang Li, Lin Cui, Sheng Li.
